# Deep convolutional GAN and hypernet-based neural architecture search for brain tumor diagnosis detection and classification

**DOI:** 10.1371/journal.pone.0352353

**Published:** 2026-07-14

**Authors:** Sreerangan Swathi, Murugasamy Rajalakshmi

**Affiliations:** 1 Department of Artificial Intelligence and Data Science, Panimalar Engineering College, Chennai, India; 2 Department of Information Technology, Coimbatore Institute of Technology, Coimbatore, India; Hanyang University - ERICA Campus, KOREA, REPUBLIC OF

## Abstract

Brain tumors are complex and life-threatening conditions that require accurate and efficient diagnostic approaches. However, existing approaches often face limitations in precision and computational efficiency, mainly due to the heterogeneous and limited nature of medical imaging datasets. Recent advancements in deep learning, mostly Neural Architecture Search (NAS) and Generative Adversarial Networks (GANs), have show significant potential for enhancing diagnostic performance. In this study, a novel framework integrating HyperNet-based Neural Architecture Search (HN-NAS) with Deep Convolutional Generative Adversarial Networks (DCGANs) is proposed for brain tumor detection and classification. The DCGAN model is employed to generate high-quality synthetic MRI images of brain lesions, thereby developing dataset diversity and mitigating the issue of limited training data. Meanwhile, HN-NAS is utilized to efficiently recognize optimal neural network architectures for accurate tumor diagnosis. The use of a HyperNetwork allows the generation of weights for multiple candidate architectures, provocatively decreasing the computational cost of architecture search and facilitating scalable model exploration. Experimental results establish that the proposed technique developments both segmentation and classification performance while maintaining computational efficiency. The findings designate that a reliable and scalable solution for real-time clinical applications can be accomplished by combining advanced NAS methods with generative models. Overall, this study establishes that integrating data augmentation with architecture optimization can suggestively improve medical imaging diagnosis.

## Introduction

The brain is the most developed organ in the human body, consisting of intricate neural networks. The neurons relate to one another and act as a response to other body organs. The development of brain cancer takes place when there is abnormal functioning of these neurons. Cancer is estimated to result in about 9.6 million deaths worldwide in 2018, and brain tumors are amongst the top causes of cancer deaths. Tumors of the brain claim close to 10% of the world deaths [1]. Brain tumors can be categorized as benign (non-cancerous) or malignant (cancerous), as well as primary or secondary. While benign tumors are usually contained and do not infiltrate surrounding tissues, malignant tumors often develop quickly, spread to other parts of the body, and offer a serious health danger. Gliomas, meningiomas, and pituitary tumors are the most prevalent forms of brain tumours. Gliomas are the most common primary brain tumors; they are made up of glial cells and are classified into four classes based on their malignancy [2]. Meningiomas are often benign, slow-growing, and limited, and they originate on the lining of the meninges or the brain’s protective layers. Compared to other tumor forms, pituitary tumors are rare and typically benign. They grow in the pituitary gland.

This is because the location, size, and shape of the tumor can all influence the symptoms. Common signs include seizures, headaches, nausea and vomiting, and difficulty in speaking or thinking coherently [3]. Biopsies, computed tomography (CT) scans, and magnetic resonance imaging (MRI) are the basic methods for diagnosing brain tumors, depending on the nature and severity of the tumor. Surgery, chemotherapy, and radiation therapy are common therapeutic techniques used to relieve symptoms, slow tumor development, and enhance patient survival rates [4,5].

Brain tumors have not been identified with specific risk factors that can be identified as being the cause of the disease in individuals of any age or sex. Some of the known risk factors are limited to hereditary disorders, exposure to ionizing radiation, and genetic factors. However, most primary brain tumors are still poorly known as regards the cause or etiology [6]. Consequently, owing to the development of medical imaging, brain cancer can now be diagnosed more effortlessly because of the emergence of modern diagnostic instruments. These have enhanced the possibility of effective therapy.

In order to decide on the right line of treatment in cases of brain tumors, proper diagnosis and classification is needed. Historically, histopathological classification has been based on traditional histological analysis of tissue samples collected by surgical means. Over the past few years, non-surgical diagnosis and characterization of brain tumors have achieved major steps forward. Magnetic resonance spectroscopy (MRS) [7], diffusion tensor imaging (DTI), magnetic resonance imaging (MRI), computed tomography (CT), and positron emission tomography (PET) are examples of advanced imaging modalities that have been used in recent research to improve the accuracy of tumor detection and staging. These methods provide more accurate and trustworthy diagnosis by offering thorough structural and functional insights.

The use of machine learning (ML) and artificial intelligence (AI) tools to analyze imaging data is one of the most recent developments in this discipline. Tumor diagnosis, classification, and prognosis prediction have all shown increased performance with these methods [8]. Nevertheless, in oncology, proper definition and diagnosis of brain tumors are difficult to achieve because of the complexity and heterogeneity of the brain tissue, both in structure and functions. Although significant advances have been made in medical imaging and diagnostic procedures, the better model design and improved interpretability of the advanced computational methods are still required [9,10].

This paper combines the utilize of DCGANs and Neural Architecture Search (NAS) based on HyperNet to identify and classify brain tumors. The suggested method takes advantage of the generative nature of DCGANs to create high-quality medical images, thus supplementing small training datasets. This improvement allows deep learning models to be more accurate when classifying different types of cancer. Moreover, the HyperNet architecture, generating model parameters dynamically depending on the input data, allows using data to select the most appropriate neural network architecture to work with certain diagnostic tasks. The main goal of the work is to establish a better patient outcome due to the faster and more precise diagnosis of a patient with the help of high-level computational methods of early detection and classification of brain tumors.

### Objective of the research

- To improve a hybrid deep learning architecture (Deep Convolutional Generative Adversarial Networks (DCGANs) and HyperNet-based Neural Architecture Search (HN-NAS)) to accurately recognize and classify brain tumors.
- To increase the range and quality of training data, synthesize high-resolution synthetic MRI images of brain tumors with DCGANs, and thereby address the limitation of small annotated medical data.
- To automatically learn good neural network structures to predict brain tumors using HyperNet-based Neural Architecture Search, and hence evading the manual engineering of models.
- To enhance the classification results and diagnostic accuracy with the help of the data augmentation and the architectural optimization methods combined in one framework.
- Reduce the complexity of computation and search time in neural architecture design, by making the efficient generation of weights possible with HyperNetworks.
- To create scalable and efficient diagnostic system that is applicable in real-time clinical practice, in the analysis of brain tumors.

### The main contribution of this research

- To incorporate HN-NAS to DCGANs to create a new paradigm of detecting and classifying brain cancers.
- Small medical datasets are considered to be a limitation, so DCGANs are applied to create high-quality synthetic MRI images of brain lesions, which enhances the diversity of training data.
- The HN-NAS objective is to effectively identify the HN architectures that offer optimal neural network models in brain tumor diagnosis.
- Lastly, architecture search can be performed with much lower computational cost by using HyperNet to compute the weights of many potential architectures, and thus explore the space of possible model configurations more quickly and scalably.

This article contains the following sections: Literature survey section provides a summary of the literature, Proposed model section specifies the technique to be followed, Result section conducts a detailed analysis of the findings, and Conclusion section makes recommendations for future research.

## Literature survey

Brain tumor detection and diagnosis has greatly improved because to machine learning (ML) algorithms and imaging technologies. According to the studies, convolutional neural networks (CNNs) are useful for analyzing MRI data in order to precisely and quickly identify malignancies. In order to progress the accuracy of analysis further, the use of ensemble learning and sophisticated feature extraction approaches is also highlighted in the studies. Different strategies, such as transfer learning and hybrid models have been considered to progress the generalization and consistency of tumor detection systems. Such developments are meant to enhance the functionality of the entire system so that the patients can get the right and timely medical attention.

Kumar et al. [11] introduced an innovative Subtractive Spatial Lightweight Convolutional Neural Network (SSLW-CNN) model, which is specifically created and trained on MRI brain imaging. The model uses new operators to simplify the classification. Class Activation Mapping is also utilized in the approach to facilitate the comprehension of the background features. The research also determines the confidence levels of the model in efficiency.

Allah et al. [12] added to the basic architecture of U-Net the Edge U-Net model which is a strong Deep Convolutional Neural Network (DCNN). They use a method that enhances better tumor detection by including MRI boundary data along with core brain MRI data. The authors indicated that the suggested framework is effective in discriminating various brain tissues, which is demonstrated in the values of Dice score. In particular, the Dice scores were 87.28% (pituitary tumors), 91.76% (gliomas), and 88.8% (meningiomas).

Filatov et al. [13] suggested the utilize of convolutional neural networks (CNNs) to classify and categorize brain tumors. The research categorized three tumor kinds based on a collection of data that comprised of non-tumor MRI data. Various architectures were used such as EfficientNetB1, EfficientNetB7, EfficientNetV2B1, and ResNet50. Among them, EfficientNet was promising with its ability to scale. The best results were obtained by EfficientNetB1 which had a 89.55% validation accuracy and 87.67% training accuracy.

To categorize brain tumors into three groups—gliomas, meningiomas, and pituitary tumors Sekhar et al. [14] developed a method based on the transfer learning methodology. To understand characteristics of brain MRI data, the authors used GoogLeNet, an already-trained Convolutional Neural Network (CNN). Several classifiers, such as Softmax, K-Nearest Neighbors (K-NN), and Support Vector Machines (SVM), were utilized to complete the classification tasks. The Harvard Medical Repository and CE-MRI Figshare datasets were utilized to train and evaluate the model. Accuracy, F1-score, and specificity measurements were utilized to assess its performance. The experimental findings display that the recommended model performs better than current techniques.

A novel hybrid deep learning (DL) method was suggested by Rasool et al. [15] to classify three kinds of human brain cancers utilizing MRI data. In their model, they used SqueezeNet as a feature extractor and applied convolutional neural network (CNN) as a classifier along with support vector machine (SVM) classifiers for classification. 98.7% classification accuracy was attained by the CNN-based models. Furthermore, their experiment showed that they could reach a general accuracy of 96.5% using an optimized version of the model: SqueezeNet. The major goal of the suggested solution is to enable the examination of brain tumors without using invasive surgery.

To overcome the limitations of the previous methods used to classify brain tumors, Bibi et al. [16] used a transfer learning approach using the InceptionV4 neural network model. They are working towards improving the quality of diagnosis, as well as reducing the time required to calculate and improve the accuracy. The model is trained using 7022 MRI images of Br35H, SARTAJ and Figshare data set. The proposed InceptionV4 model is effective in that it differentiates normal brain images and classifies brain cancers into three categories with the help of transfer learning. The model has proved to be effective since it has accomplished a classification accuracy of 98.7% in recognizing brain tumors.

Farzamnia et al. [17] enhanced a novel method of distinction between benign and malignant brain tumors on MRI images with the use of contourlet transform and a time-adaptive self-organizing map optimized by the Whale Optimization Algorithm. To diagnose and treat brain tumors, it is important to categorize. The given method is highly accurate and outperforms the existing ones when it comes to classifying MRI brain images. The experimental evidence indicates that the approach has an effective execution rate and 98.5% classification accuracy given a set of test cases.

Ismael et al. [18] implemented a Residual Network (ResNet) to develop a brain tumor classification system. A benchmark dataset including 3,064 brain MRI scans representing three distinct tumor types gliomas, meningiomas, and pituitary tumors was used to test the model. When it comes to classifying brain tumors, their deep learning model outperforms previous approaches. Brain tumors can be efficiently classified by the optimized deep learning system, which also enhances performance.

Rehman et al. [19] trained CNN systems, AlexNet, GoogLeNet and VGGNet, to improve a brain cancer classification system, which included meningiomas, glioma, and pituitary tumors. The Figshare dataset that consists of MRI slices of brain tumors was preprocessed and normalized using the transfer learning techniques. They augmented the data utilized to train the models with data augmentation techniques on the MRI slices, which amplified the number of training samples and reduced overfitting, thus enhancing the soundness of the analysis. Optimizing the VGG16 architecture to reach a 98.69% classification and detection accuracy.

An improved computer-aided diagnostic technique for classifying brain cancer was presented by Lee et al. [20]. To lessen noise in magnetic resonance imaging data and enhance deep learning models’ capacity for generalization, they used GridMask in conjunction with Gaussian filtering. They then enhanced a new method known as Patterned-GridMask to get beyond the drawbacks of traditional GridMask, which can mask essential tumor areas. In their trials, four deep learning models were assessed: TresNet-M, EfficientNetV2-M, MaxViT-B, and ViT-B/16. Performance was improved by up to 6% when Patterned-GridMask was utilized. The top-performing model has an accuracy of 97.74% and an F1-score of 97.75%.

In order to progress feature extraction and enable precise brain tumor segmentation, Liu et al. [21] suggested a CNN-based architecture that includes Gaussian filtering as a preprocessing step. According to several studies, which include those that use both MRI scans of tumor-affected and healthy brains to train neural networks, significant accuracy in classification of brain cancer has been achieved. Moreover, morphological operations are useful in improving the accuracy of classification because they eliminate noise in segmented images, thus allowing the effective distinction of normal and dysfunctional brain tissues.

### Limitations of existing work

The current research still has a number of shortcomings, despite notable progresses in deep learning and medical imaging methods for brain tumor identification. A lot of methods are based on rather small and homogeneous datasets, which reduces the generalizability and robustness of the trained models. Moreover, inconsistencies emerge with differences in imaging modalities, scanner settings, and acquisition conditions, which have negative impacts on model performance.

Moreover, most of the currently available techniques largely concentrate on single-modality data (e.g., MRI), thus not taking advantage of complementary information provided by multimodal data (e.g., CT or PET scans). The natural heterogeneity of brain tumors such as size, shape, and texture cannot be well modeled by traditional architectures, and thus classification is poor on challenging cases.

The other significant constraint is that the deep learning models lack interpretability and transparency, making it challenging to implement them in clinical practice. In addition, the conventional design of neural architectures is deeply based on manual tuning, which leads to higher computational cost and poor model design. Lastly, the annotated medical data is an important bottleneck that restricts the usefulness of supervised learning strategies.

### Research gap

- Although deep learning-based brain tumor detection has made tremendous progress, there are still a number of gaps that should be addressed. Current approaches might be based on small and unimodal data, which limits their applicability to different clinical conditions. In addition, the majority of methods do not have effective mechanisms to deal with tumor heterogeneity in terms of size, shape and texture.
- Also, manual design of neural network architectures can frequently lead to poor performance and higher computational cost. The existing models also have low interpretability, which decreases its clinical credibility and applicability. Additionally, there is no annotated medical data, which is also an obstacle to quality model training.
- To overcome these obstacles, an integrated framework that combines robust feature learning, architectural optimization, and data augmentation is required to rise the clinical reliability, scalability, and accuracy of brain tumor diagnosis.

## Proposed model

**Fig 1:**
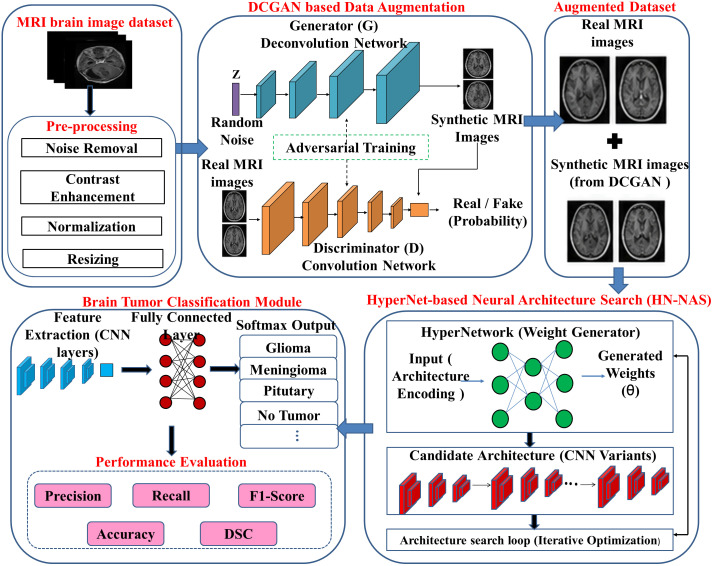
Block diagram of the recommended technique.

This paper combines DCGANs and HN-NAS in the effort to introduce a new paradigm to enhance brain tumor detection and classification. DCGANs can help meet the limitation of limited medical data by creating high-quality synthetic MRI images of the lesions in the brain. To accurately diagnose brain cancers, HN-NAS optimizes the selection of neural network designs. By employing a HyperNet to calculate the weights of a collection of alternative designs, the search cost for the architecture is significantly reduced, enabling much faster and more scalable exploration of possible model choices. The block diagram of the DCGAN-HN-NAS architecture is display in Fig 1.

The suggested design, which successfully identifies brain tumors by combining HyperNet-driven Neural design Search (HN-NAS) with DCGAN-based data augmentation, is display in Fig 1. First, MRI brain images are initially processed in a systematic way; noise is removed, contrast is boosted, images are normalized and resized. The processed images are then inputted into a DCGAN, whereby the generator generates realistic synthetic MRI images and the discriminator judges their quality by means of adversarial learning, thus increasing the diversity of the datasets. The augmented dataset is later employed in the HN-NAS module, whereby a HyperNetwork is employed to produce weights of numerous candidate architectures and determine the best model by evaluating the architectures through iterative evaluation. Lastly, the chosen architecture can classify tumors (glioma, meningioma, pituitary, or no tumor) and the performance of the model is measured in terms of accuracy, precision, recall, F1-score, and Dice Similarity Coefficient (DSC).

### Dataset

In this work, 3,264 T1-weighted contrast-enhanced MRI brain images were obtained from a publically available dataset. The dataset includes 500 images of normal brains, 926 glioma images, 937 meningioma images, and 901 pituitary tumor images. These images were taken from numerous anatomical planes, including axial, coronal, and sagittal perspectives, which increased the dataset’s diversity and resilience. To facilitate model building and evaluation, the dataset was separated into training and testing subsets. The training set consists of 827 pituitary tumor images, 822 meningioma images, 826 glioma images, and 395 normal brain images. The testing set includes 74 pituitary tumor images, 115 meningiomas, 100 gliomas, and 105 normal brain images. This is a balanced and well-structured data that helps to form a strong classification model since it has sufficient representation of diverse tumor types and normal cases [22]. Table 1 provides a detailed description of the dataset utilized in this study.

**Table 1:** Dataset Description.

Tumor Types	Testing Images	Training Images
**Pituitary Gland Tumor**	74	827
**Meningioma**	115	822
**Healthy Brain**	105	395
**Glioma**	100	826

### Image pre-processing

This study introduces a new hybrid contrast enhancement method based on functions of kurtosis and absolute mean deviation. By greatly increasing the contrast of brain tumors, the suggested method progresses the quality of brain images for subsequent processing. Let D stand for the database of brain tumors, and let N be the input for the UXV dimensions of the brain image [23].

Consider the input image X, which has dimensions of N×M by Δ and was taken from the brain tumor database. Let nbe the entire quantity of pixels in this image, and let $x_{i}$ be the value of each pixel. In Eq (1), the Absolute Mean Deviation (AMD) is clear, and in (2), kurtosis is defined.


$$\begin{array}{c} AD=\frac{\text{1}}{n}\sum_{i=1}^{n}\left|x_{i}-\phi \left(X\right)\right| \end{array}$$ (1)

$$\begin{array}{c} SK=\frac{\frac{\text{1}}{S}\overset{m}{\underset{k=1}{\sum }}{\|x_{i}-X\|}^{\text{3}}}{\left(n-1\right)*{\|s\|}^{\text{3}}} \end{array}$$ (2)

In this case, AD stands for the image’s AMD, ϕ(X) for the dataset’s average mean (Δ) and S for the standard deviation($\sqrt{\frac{\overset{n}{\underset{i=1}{\sum }}{\left(x_{i}-\overset{―}{x}\right)}^{\text{2}}}{n}}$). X stands for the mean (average) of the information ideas. The last image alteration using these valuesis $I_{\text{1}}=AD\left(i\right)+X$ and $IF=I_{\text{1}}-SK\left(i\right)$ where i denotes the image pixels and IF signifies the final modified image. This process is applied to all authorized datasets before training the learning models.

### Deep convolutional GAN

**Fig 2:**
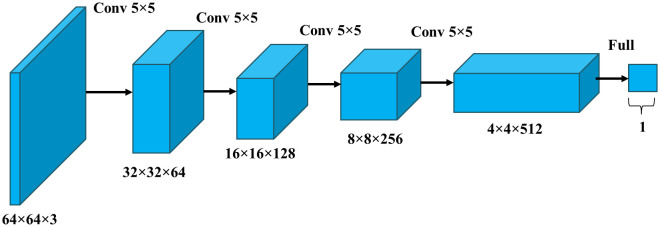
Layered Architecture of DCGAN.

A DCGAN uses the advantages of GANs, CNNs, and deep learning to increase the precision of identifying and categorizing brain cancers in medical imaging, such as MRI images [24]. This method uses a competitive zero-sum game among two neural networks, the discriminator (D) and the generator (G), to increase classification performance and the model’s capacity to produce realistic synthetic brain tumor images. Fig 2 shows the DCGAN’s architecture.

**Generator network (g):** G(z)

- The generator is responsible for creating synthetic MRI images that closely resemble actual brain tumor scans. By passing random noise sampled from a latent space through a series of transposed convolutional layers, it generates high-resolution images.
- It is expected that the generator will produce a synthetic image that the discriminator can differentiate from real images.

The loss of the producer is defined as follows in Eq (3):


$$\begin{array}{c} L_{G}=-{𝔼}_{z\sim \;p_{z}\left(z\right)}\left[log\left(D\left(G\left(z\right)\right)\right)\right] \end{array}$$ (3)

Where D(G(z)) is the likelihood that the generated image is authentic, as the discriminator had anticipated.

**Discriminator Network (D)**

- The discriminator separates created images from actual brain tumor images in the dataset, whereas the generator produces artificial images. Both feature extraction and binary classification (fake or real) employ convolutional layers.
- The discriminator aims to accurately differentiate among real and fake images.

The discriminator loss is defined as Eq (4):


$$\begin{array}{c} L_{D}=-{𝔼}_{x\sim \;p_{data}\left(x\right)}\left[log\left(D\left(x\right)\right)\right]-{𝔼}_{z\sim \;p_{z}\left(z\right)}\left[log\left(1-D\left(G\left(z\right)\right)\right)\right] \end{array}$$ (4)

Where D(x) is the discriminator’s prediction that x is genuine, and x is a real image from the training set.

**Total loss**

The GAN optimization is carried out by alternately training both networks. The model’s total objective function is given below:


$$\begin{array}{c} \underset{G}{min}\;\underset{D}{max}\;L\left(G,D\right)={𝔼}_{x\sim \;p_{data}\left(x\right)}\left[log\;D\left(x\right)\right]+{𝔼}_{z\sim \;p_{z}\left(z\right)}\left[log\left(1-D\left(G\left(z\right)\right)\right)\right] \end{array}$$ (5)

This is the goal of the generator, which the discriminator tries to maximize.

**Tumor classification**

Once the GAN has been trained, the discriminator or a different CNN classifier is fine-tuned to classify actual brain MRI images into categories (e.g., benign and malignant) according to features that have been learned during the adversarial training. The DCGAN model can complement the classification and diagnostic process by focusing on the finer and discriminative details of brain tumor images using the adversarial learning mechanism.

### Hypernet-based Neural Architecture Search (HN-NAS)

The HyperNet-based Neural Architecture Search (NAS) framework to detect, recognize, and classify brain cancers using a HyperNetwork to create weights to the target neural network is utilized to detect tumor. The method allows the model to be more flexible since the network architecture can be auto-tuned in response to accuracy or loss and does not need human intervention. NAS mechanism finds the best architectures by searching through potential combinations of layers, neurons, and activation functions [25]. This method is especially useful when it comes to automated design of effective deep learning models to detect and classify brain tumors.

HyperNet-based NAS consists of two important elements:

- HyperNetwork: A neural network that generates the weights of another neural network (the target model).
- Search Algorithm: An optimization approach, similar to reinforcement learning or evolutionary algorithms, explores various architectural configurations to recognize the most effective model for brain tumor classification.

**Objective function for architecture search:**


$$\begin{array}{c} \mathrm{\backslash stackrel}min\alpha ,\theta L\left(f\left(x;\alpha ,\theta \right),y\right) \end{array}$$ (6)

Where:

- For classification problems, L(·) stands for the loss function, such as cross-entropy.
- f(x;α,θ) is the target network with architecture parameters α and model weights θ.
- x is the input data (e.g., brain tumor images), and y is the ground truth (e.g., tumor classification labels).

**Hyper network output**


$$\begin{array}{c} \theta =g\left(\alpha ;\phi \right) \end{array}$$ (7)

Where:

- g(·) is the HyperNetwork with its own parameters ϕ.
- α represents the architectural parameters (like layer configurations) that the HyperNetwork uses to generate the target model weights θ.

**Update Rule for NAS (using Reinforcement Learning as an example)**


$$\begin{array}{c} \alpha_{t+1}=\alpha_{t}+\eta \cdot \nabla_{\alpha }R\left(\alpha_{t}\right) \end{array}$$ (8)

Where:

- $\alpha_{t}$ represents the architecture at iteration t.
- $R\left(\alpha_{t}\right)$ is the reward (e.g., classification accuracy).
- η is the learning rate for updating the architecture.

**Cross-entropy loss for tumor classification**


$$\begin{array}{c} L_{CE}\left(y,\hat{y}\right)=-\sum_{i=1}^{N}y_{i}log\left({\hat{y}}_{i}\right) \end{array}$$ (9)

Where:

- The correct label is represented by y, while the anticipated likelihood for each class (such as tumor kind) is specified by $\hat{y}$.
- N Is the number of classes.

By combining hypernetwork-based weight generation with neural architecture search (NAS) optimization, this approach can lead to a more effective and potentially more accurate brain tumor classification model.

Initialize GAN_model

$G_{\theta_{g}},D_{\theta_{d}}\sim \;N\left(0,\sigma^{\text{2}}\right)$

Initialize HyperNet_model

$H_{\theta_{h}}\sim \;N\left(0,\overset{\text{2}}{\underset{h}{\sigma }}\right)$

load brain_tumor_data

$\left\{\left(X_{i},Y_{i}\right)\right\}\text{\textbackslash{}hspace\{0.17em}\;\;\}for\text{\textbackslash{}hspace\{0.17em}\;\;\;\}i=1,2,...,N$

while not GAN_model.converged:

for each batch of brain_tumor_data:

$\hat{X}=G\left(z\right)\text{\textbackslash{}hspace\{0.17em}\;\}where\text{\textbackslash{}hspace\{0.17em}\;\;\}z\sim \;p_{z}$

$L_{D}=-E_{x\sim \;p_{data}}\left[logD\left(x\right)\right]-E_{z\sim \;p_{z}}\left[log\left(1-D\left(G\left(z\right)\right)\right)\right]$

Update discriminator weights θ:

$\theta_{d}\leftarrow \theta_{d}-\eta \nabla_{\theta_{d}}L_{D}$

$L_{G}=-E_{z\sim \;p_{z}}\left[logD\left(G\left(z\right)\right)\right]$

Update generator weights

$\theta_{d}\leftarrow \theta_{d}-\eta \nabla_{\theta_{d}}L_{G}$

update GAN_model weights

while not HyperNet_model.converged:

for $P_{k}=Performance\left(A_{k},X,Y\right)$

evaluate architecture on brain_tumor_data

$Performance\text{\textbackslash{}hspace\{0.17em}\;\;\}List=\{P_{\text{1}},P_{\text{2}},....,P_{m}\}$

select $Top\text{\textbackslash{}hspace\{0.17em}\;\}Architecture=\{A_{top1},A_{top2},....,A_{topn}\}$

for each selected architecture of $A_{top}\leftarrow Train\left(A_{top},X_{tain},Y_{train}\right)$:

train architecture on training_data

evaluate architecture on $Performance_{val}=Evaluate\left(A_{top},X_{val},Y_{val}\right)$

for each patient of $\hat{Y}=A_{best}\left(X_{test}\right)$:

classify_tumor = best_model.predict(patient_MRI)

if classify_tumor is positive:

diagnosis = “ $\hat{Y}=1\text{\textbackslash{}hspace\{0.17em}\;\}\left(Tumor\text{\textbackslash{}hspace\{0.17em}\;\}Detected\right)$”

else:

diagnosis = “ $\hat{Y}=0\text{\textbackslash{}hspace\{0.17em}\;\;\}\left(No\text{\textbackslash{}hspace\{0.17em}\;\}Tumor\text{\textbackslash{}hspace\{0.17em}\;\}Detected\right)$”

print diagnosis

Where θg and θd are the weights for the generator and discriminator separately, initialized from a normal dissemination with mean 0 and variance σ2 and θh are the weights of the HyperNet. N is the number of examples, Xi are the MRI images, and Yi ∈{0,1} (0: No Tumor, 1: Tumor Detected).

## Result

### Implementation setup

A high-performance computer system with an Intel Core i9 (12th Generation) processor, 32 GB of RAM, and an NVIDIA GTX 1060 with 8 GB of RAM was utilized for all of the trials. This was implemented in Python using deep learning packages like TensorFlow/Keras and PyTorch to guarantee effective model training and scalability. The proposed DCGAN-HN-NAS model is trained with a well-chosen set of hyperparameters to guarantee the stable training and optimal performance. The learning rate is adjusted to 0.001 to trade-off between training stability and the convergence rate. The batch size is 32, which is used to effectively use the computational resources and still achieve good generalization. The model is trained over 50–100 epochs, depending on the convergence behavior, to avoid overfitting and guarantee satisfactory learning. The Adam optimizer is used because it has adaptive learning and it is efficient in dealing with sparse gradients. In the classification task, the cross-entropy loss is employed, whereas in the context of the GAN, adversarial loss is employed to direct the networks of the generator and discriminator to generate realistic synthetic MRI images.

### Virtual intelligent laboratory environment

The suggested system is deployed in the Virtual Intelligent Laboratory (VIL) that offers a cloud-based and scalable platform to execute the deep learning models. The laboratory environment enables remote access, computation with GPUs, and interactive experimentation, based on Python-based frameworks like TensorFlow and PyTorch. The system is available via a web-based interface or on cloud platforms that allow uploading datasets, training models, and evaluating their performance in real-time. Such virtual environment improves the reproducibility, scalability, and accessibility of the suggested DCGAN-HN-NAS framework.

### Performance metrics

The model can be assessed utilizing performance metrics with regards to the various parameters discussed below. For instance,

**Precision**

The measure of accuracy of positive predictions is called precision. It is determined by dividing the number of predicted positive results that were correct by all the positive results predicted by the model.


$$\begin{array}{c} Precision=\frac{True\text{\textbackslash{}hspace\{0.17em}}{P}ositives\}True\text{\textbackslash{}hspace\{0.17em}Positives+False\text{\textbackslash{}hspace\{0.17em}\}Positives\} \end{array}$$ (10)

**Recall**

In classification problems, recall is a performance indicator that assesses how well a model finds all pertinent examples in a dataset. It shows the percentage of real positive cases that the model accurately predicted.


$$\begin{array}{c} Recall=\frac{True\text{\textbackslash{}hspace\{0.17em}}{P}ositives\}True\text{\textbackslash{}hspace\{0.17em}Positives+False\text{\textbackslash{}hspace\{0.17em}\}Negatives\} \end{array}$$ (11)

**F1-Score**

Precision and recall are combined into a single performance metric called the F1-score. Recall is the percentage of correctly predicted positive observations among all actual positive cases, whereas precision is the percentage of correctly predicted positive observations among all projected positive instances. The F1-score, which offers a fair assessment of the model’s overall performance, is computed as the harmonic mean of precision and recall.


$$\begin{array}{c} F1-Score=2\times \frac{Precision\times Recall}{Precision+Recall} \end{array}$$ (12)

**Accuracy**

One of the parameters that are important to determine the effectiveness of an organization model is accuracy. It denotes the percentage of cases in the dataset that are correctly classified, including true positives and true negatives.


$$\begin{array}{c} Accuracy=\frac{TP+TN}{TP+TN+FP+FN} \end{array}$$ (13)

**Deice Similarity Coefficient (DSC)**

DSC is a popular measure to evaluate the similarity of two sets in image segmentation and classification tasks.


$$\begin{array}{c} DSC=\frac{2\left|A\cap B\right|}{\left|A\right|+\left|B\right|} \end{array}$$ (14)

### Precision analysis

**Fig 3:**
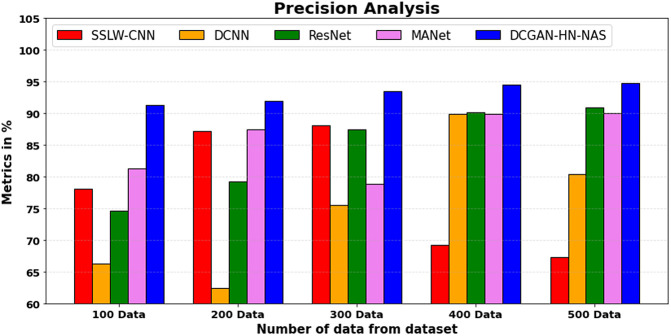
Precision Analysis for DCGAN- HN-NAS method.

The precision analysis of the proposed DCGAN–HN–NAS method, in comparison with existing models across numerous dataset sizes, is presented in Table 2 and Fig 3. The proposed method outperforms SSLW-CNN (78.11%), DCNN (66.33%), ResNet (74.56%), and MANet (81.23%), accomplishing a precision of 91.35% for 100 samples. For 200 samples, DCGAN–HN–NAS maintains greater performance with a precision of 91.99%, while the other models range among 62.37% and 87.44%. At 300 samples, the proposed technique further progresses to 93.45%, associated to SSLW-CNN (88.15%), DCNN (75.56%), ResNet (87.44%), and MANet (78.91%). For 400 samples, it accomplishes 94.55%, slightly higher than MANet (89.91%), ResNet (90.11%), DCNN (89.93%), and SSLW-CNN (69.17%). Lastly, at 500 samples, DCGAN–HN–NAS attains the highest precision of 94.78%, outperforming SSLW-CNN (67.34%), DCNN (80.45%), ResNet (90.91%), and MANet (89.99%). Overall, the results clearly establish that the proposed technique consistently accomplishes the highest precision across all dataset sizes, demonstrating enhanced feature learning and reduced false positive rates.

**Table 2:** Precision Analysis of the DCGAN-HN-NAS Method.

Number of data from Dataset	SSLW-CNN	DCNN	ResNet	MANet	DCGAN- HN-NAS
**100**	78.11	66.33	74.56	81.23	91.35
**200**	87.23	62.37	79.19	87.44	91.99
**300**	88.15	75.56	87.44	78.91	93.45
**400**	69.17	89.93	90.11	89.91	94.55
**500**	67.34	80.45	90.91	89.99	94.78

### Recall analysis

**Fig 4:**
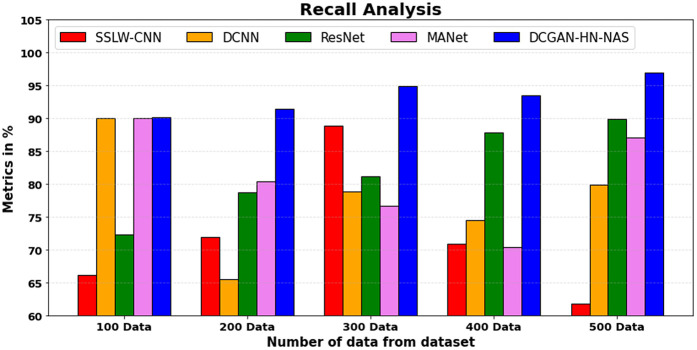
Recall Analysis for DCGAN- HN-NAS method.

Table 3 and Fig 4 present the recall performance comparison of the proposed DCGAN–HN-NAS technique against existing models across different dataset sizes. The proposed method outperforms SSLW-CNN (66.12%), DCNN (89.98%), ResNet (72.33%), and MANet (89.98%), accomplishing a recall of 90.16% for 100 samples. At 200 samples, DCGAN–HN-NAS further progresses to 91.45%, while the competing approaches range from 65.56% to 80.44%. For 300 samples, the proposed technique reaches 94.91%, associated to 88.81% (SSLW-CNN), 78.91% (DCNN), 81.17% (ResNet), and 76.66% (MANet). At 400 samples, it maintains strong performance with a recall of 93.45%, whereas the other models vary among 70.44% and 87.78%. Lastly, at 500 samples, the DCGAN–HN-NAS method accomplishes the highest recall of 96.98%, suggestively outperforming SSLW-CNN (61.81%), DCNN (79.91%), ResNet (89.91%), and MANet (87.13%). Overall, the results clearly demonstrate that the proposed technique consistently accomplishes greater recall across all dataset sizes, highlighting its strong ability to correctly recognize positive tumor cases.

**Table 3:** Recall Analysis for the DCGAN-HN-NAS Method.

Number of data from Dataset	SSLW-CNN	DCNN	ResNet	MANet	DCGAN- HN-NAS
**100**	66.12	89.98	72.33	89.98	90.16
**200**	71.91	65.56	78.71	80.44	91.45
**300**	88.81	78.91	81.17	76.66	94.91
**400**	70.91	74.45	87.78	70.44	93.45
**500**	61.81	79.91	89.91	87.13	96.98

### Accuracy analysis

**Fig 5:**
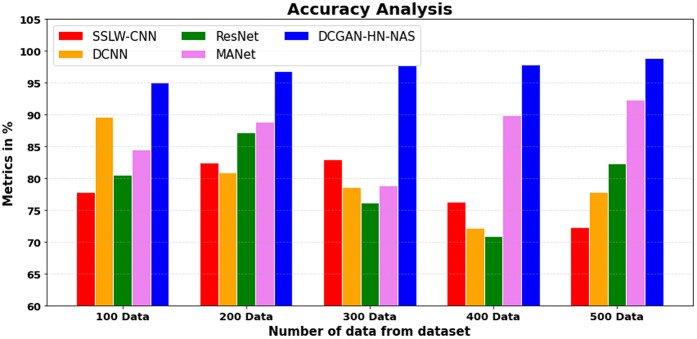
Accuracy Analysis for DCGAN- HN-NAS method.

The accuracy analysis of the proposed DCGAN-HN-NAS method, in comparison with existing techniques across various dataset sizes, is presented in Table 4 and Fig 5. The proposed technique accomplishes an accuracy of 94.98% for 100 samples, outperforming SSLW-CNN (77.83%), DCNN (89.64%), ResNet (80.55%), and MANet (84.45%). At 200 samples, the accuracy further improves to 96.81%, compared to 82.45%, 80.91%, 87.23%, and 88.82% for the respective models. For 300 samples, DCGAN-HN-NAS reaches 97.71%, while the other models exhibit lower performances of 82.92%, 78.54%, 76.17%, and 78.91%. At 400 samples, the proposed technique maintains a high accuracy of 97.91%, significantly surpassing SSLW-CNN (76.29%), DCNN (72.19%), ResNet (70.93%), and MANet (89.88%). Lastly, for 500 samples, the DCGAN-HN-NAS method accomplishes the highest accuracy of 98.91%, associated to SSLW-CNN (72.27%), DCNN (77.88%), ResNet (82.28%), and MANet (92.34%). Overall, the results show that the suggested technique continuously outperforms all baseline models and improves accuracy as the dataset size increases, validating its efficacy and robustness in brain tumor classification.

**Table 4:** Analysis of Accuracy for the DCGAN-HN-NAS Approach.

Number of data from Dataset	SSLW-CNN	DCNN	ResNet	MANet	DCGAN- HN-NAS
**100**	77.83	89.64	80.55	84.45	94.98
**200**	82.45	80.91	87.23	88.82	96.81
**300**	82.92	78.54	76.17	78.91	97.71
**400**	76.29	72.19	70.93	89.88	97.91
**500**	72.27	77.88	82.28	92.34	98.91

### F1-score analysis

**Fig 6:**
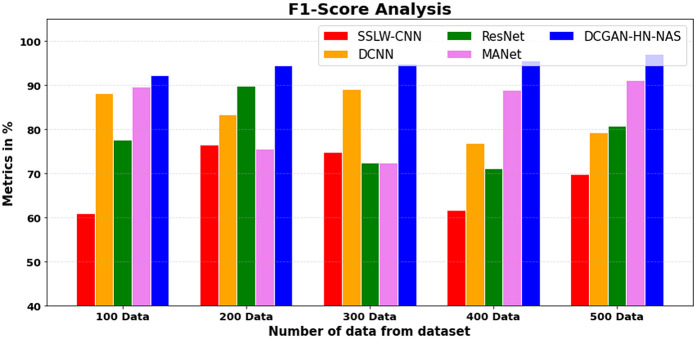
F1-Score Analysis for DCGAN- HN-NAS method.

The F1-score analysis presented in Table 5 and Fig 6 founds that the proposed DCGAN–HN-NAS method consistently outperforms all comparative models across different dataset sizes. For 100 samples, the proposed technique achieves an F1-score of 92.34%, connected to SSLW-CNN (60.91%), DCNN (88.16%), ResNet (77.55%), and MANet (89.76%). At 200 samples, DCGAN–HN-NAS further advances to 94.56%, while SSLW-CNN, DCNN, ResNet, and MANet achieve 76.45%, 83.37%, 89.91%, and 75.56%, consistently. For 300 samples, the proposed method records 94.81%, outperforming DCNN (89.18%), SSLW-CNN (74.88%), ResNet (72.34%), and MANet (72.34%). At 400 samples, DCGAN–HN-NAS reaches 95.67%, whereas MANet, DCNN, ResNet, and SSLW-CNN achieve 88.88%, 76.91%, 71.11%, and 61.66%, consistently. Finally, at 500 samples, the proposed technique attains the highest F1-score of 97.11%, provocatively surpassing MANet (91.23%), ResNet (80.81%), DCNN (79.33%), and SSLW-CNN (69.91%). These results clearly designate that the proposed technique maintains a greater balance amongst precision and recall across all dataset sizes, representing its robustness and efficiency in brain tumor classification.

**Table 5:** F1-Score Analysis for DCGAN- HN-NAS technique.

Number of data from Dataset	SSLW-CNN	DCNN	ResNet	MANet	DCGAN- HN-NAS
**100**	60.91	88.16	77.55	89.76	92.34
**200**	76.45	83.37	89.91	75.56	94.56
**300**	74.88	89.18	72.34	72.34	94.81
**400**	61.66	76.91	71.11	88.88	95.67
**500**	69.91	79.33	80.81	91.23	97.11

### Dice similarity coeffiecient

**Fig 7:**
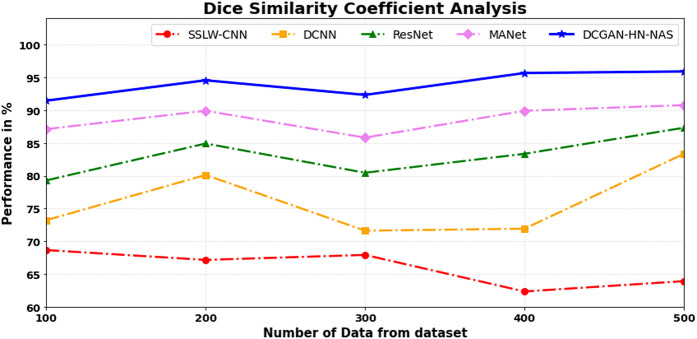
DSC Analysis for DCGAN- HN-NAS method.

The Dice Similarity Coefficient (DSC) analysis presented in Table 6 and Fig 7 demonstrates the greater segmentation performance of the proposed DCGAN–HN-NAS method associated to existing models across all dataset sizes. For 100 samples, the proposed method accomplishes a DSC of 91.45%, outperforming SSLW-CNN (68.65%), DCNN (73.21%), ResNet (79.28%), and MANet (87.11%). As the dataset size rises to 200, the DSC further progresses to 94.56%, while the comparative models accomplish 67.15%, 80.11%, 84.91%, and 89.91%, correspondingly. At 300 samples, the proposed technique maintains strong performance with a DSC of 92.33%, exceeding SSLW-CNN (67.91%), DCNN (71.61%), ResNet (80.45%), and MANet (85.81%). For 400 samples, the DSC rises to 95.67%, whereas the other models accomplish 62.33%, 71.91%, 83.34%, and 89.91%. Lastly, at 500 samples, the proposed DCGAN–HN-NAS attains the highest DSC of 95.91%, suggestively outperforming SSLW-CNN (63.91%), DCNN (83.34%), ResNet (87.32%), and MANet (90.77%). In general, the findings distinctly establish that the proposed technique always achieves the highest segmentation accuracy in all sizes of the dataset, which indicates its strength and more significant ability to overlap the space.

**Table 6:** DSC Analysis for DCGAN- HN-NAS method.

Number of data from Dataset	SSLW-CNN	DCNN	ResNet	MANet	DCGAN- HN-NAS
**100**	68.65	73.21	79.28	87.11	91.45
**200**	67.15	80.11	84.91	89.91	94.56
**300**	67.91	71.61	80.45	85.81	92.33
**400**	62.33	71.91	83.34	89.91	95.67
**500**	63.91	83.34	87.32	90.77	95.91

### Execution time analysis

**Fig 8:**
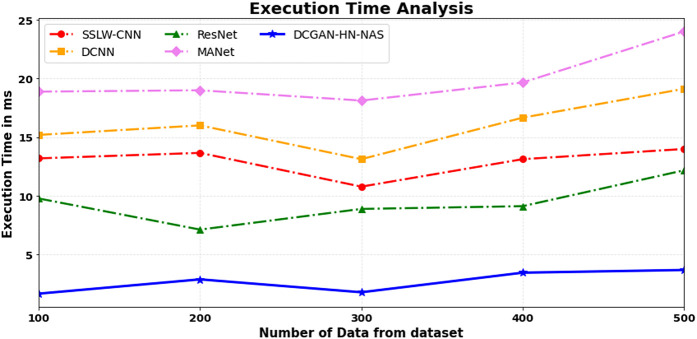
Analysis of execution time for the DCGAN-HN-NAS method.

The execution time analysis presented in Table 7 and Fig 8 establishes that the proposed DCGAN-HN-NAS method consistently accomplishes the lowest computational time across all dataset sizes associated to existing approaches. For 100 samples, the proposed method necessitates only 1.654 ms, whereas SSLW-CNN, DCNN, ResNet, and MANet need 13.187 ms, 15.187 ms, 9.765 ms, and 18.876 ms, correspondingly. Likewise, for 200 samples, the proposed technique records 2.871 ms, which is suggestively lower than SSLW-CNN (13.654 ms), DCNN (15.998 ms), ResNet (7.113 ms), and MANet (18.991 ms). At 300 samples, DCGAN-HN-NAS accomplishes 1.771 ms, associated to 10.776 ms, 13.118 ms, 8.876 ms, and 18.115 ms for the respective models. For 400 samples, the proposed method records 3.441 ms, while SSLW-CNN, DCNN, ResNet, and MANet exhibit higher execution times of 13.117 ms, 16.654 ms, 9.111 ms, and 19.654 ms, correspondingly. Finally, at 500 samples, DCGAN-HN-NAS maintains superior efficiency with 3.671 ms, whereas the competing methods require 13.987 ms, 19.114 ms, 12.165 ms, and 23.998 ms, respectively. Overall, the results clearly designate that the proposed technique significantly reduces computational overhead while maintaining scalability across increasing dataset sizes.

**Table 7:** Analysis of execution time for the DCGAN-HN-NAS algorithm.

Number of data from Dataset	SSLW-CNN	DCNN	ResNet	MANet	DCGAN- HN-NAS
**100**	13.187	15.187	9.765	18.876	1.654
**200**	13.654	15.998	7.113	18.991	2.871
**300**	10.776	13.118	8.876	18.115	1.771
**400**	13.117	16.654	9.111	19.654	3.441
**500**	13.987	19.114	12.165	23.998	3.671

### Comparative methods

Subtractive Spatial Lightweight Convolutional Neural Network (SSLW-CNN) [11]: This approach is applied to MRI-based brain images and introduces novel operators designed to simplify classification.

Deep Convolutional Neural Network (DCNN) [12]: By integrating critical information from brain MRIs with knowledge of MRI boundaries, the model efficiently diagnoses cancer.

Residual Network (ResNet) [18]: The proposed deep learning model outperforms existing approaches in identifying brain cancer. Its innovative architecture significantly enhances performance and effectively optimizes the classification of brain tumors.

Multi-level Attention Network (MANet) [26]: Using the 3,064 T1W-CE MRI datasets, the proposed architecture integrates MANet, which combines cross-channel and spatial attention mechanisms.

### Statistical analysis

The performance and reliability of the proposed method are essential and should be determined by statistical analysis. Precision, recall, accuracy, F1-score, and Dice Similarity Coefficient (DSC) are among the important performance indicators used in this study’s thorough review. These are numerical measures of how well the model performs when applied to data sets of varying sizes. Moreover, the effectiveness and superiority of the proposed DCGAN-HN-NAS strategy are verified by comparing them with the related baseline models. The model’s dependability and generalizability are guaranteed by the use of cross-validation procedures, and its stability and predictive power are further illuminated by the trends and variances in performance measures.

**Fig 9:**
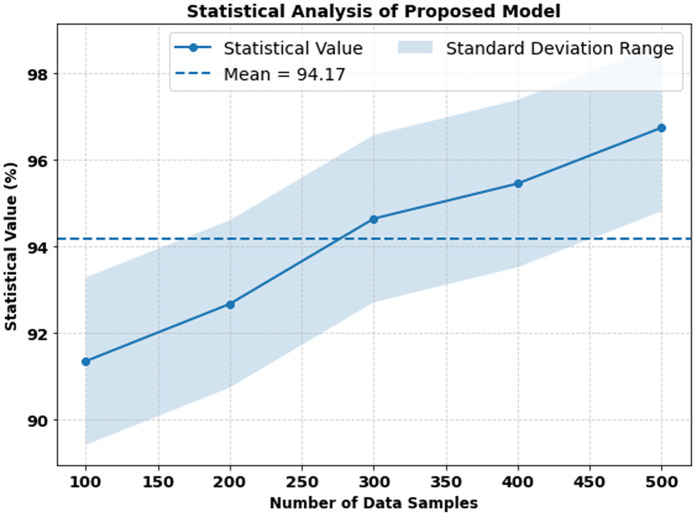
Statistical Analysis of proposed Model.

Fig 9 demonstrates the statistical dynamics of the recommended approach in relation to the changes in data samples, which show the consistency and stability of the approach. The plotted values are on a constant increasing trend which points to improved performance with increase in the size of the dataset. The average (94.17) is an indicator of the good performance of the model, and the shaded area of standard deviation shows that there is not much variation in the observations. This designates that the model has reliable and consistent results, which proves its strength and the ability to generalize.

**Fig 10:**
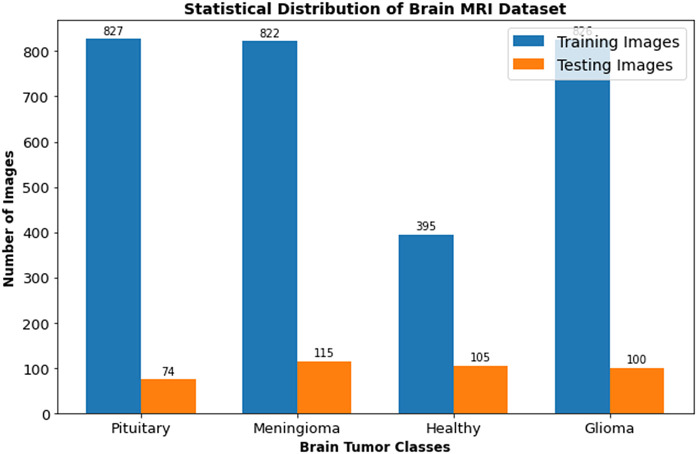
Statistical Distribution of Brain MRI Dataset.

The statistical distribution of training and testing images for several types of brain tumors, such as pituitary, meningioma, glioma, and normal (healthy) instances, is shown in Fig 10. The dataset is also fairly balanced in its training samples, where more images are used in the model learning process than in testing. The sample sizes used in testing are also smaller and evenly spread across all classes to ensure a good evaluation. This equal distribution aids successful training of models as well as ensuring equal performance evaluation among various types of tumors.

**Fig 11:**
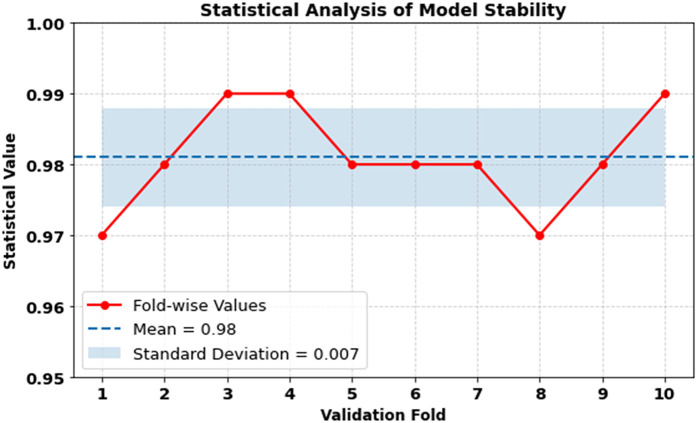
Statistical Analysis of Model Stability.

Fig 11 shows the statistical values of cross-validation by fold-wise, which indicates the stability and the consistency of the proposed method. The findings are also very close to the mean value of 0.98 with a very low standard deviation of 0.007 that shows little variation among folds. Despite the observed small fluctuations, the general trend shows that the model is reliable in its performance irrespective of data partitioning. This validates the strength and good generalization ability of the recommended method.

**Fig 12:**
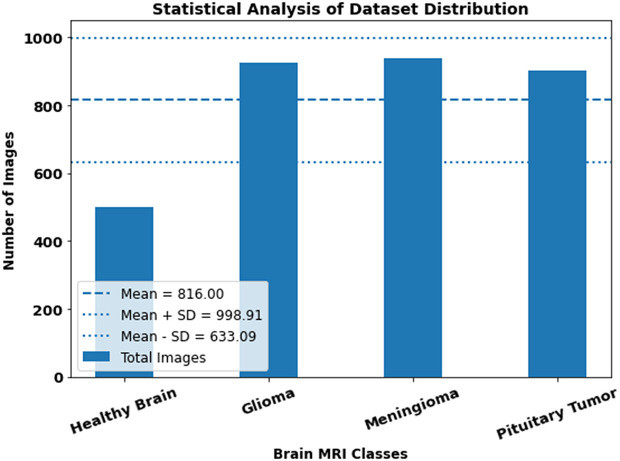
Statistical Analysis of Dataset Distribution.

Fig 12 depicts the statistical distribution of the total number of images in the dataset that belong to various categories. The mean of 816 images per class and the upper and lower limits (mean standard deviation) indicate the dispersion and variability of the data distribution. Most categories are in this range meaning that the data set is relatively balanced, however, minor changes also exist. This distribution promotes consistent learning and minimizes the chance of imbalance in the classroom by making sure that the model is trained on diverse enough samples.

### Discussion

The experimental results clearly show that the recommended DCGAN-HN-NAS technique improves the performance of brain tumor identification and classification. The improved performance can be mainly explained by the synergetic combination of data augmentation with DCGAN and automated architecture optimization with HyperNet-based NAS. To start with, the reduction of the error rate in all the dataset sizes implies that the proposed model contributes significantly to minimizing false positive errors. This can primarily be attributed to the fact that DCGAN can produce realistic and diversified synthetic MRI samples that add variety to the training distribution and allow the model to be more sensitive in discriminating delicate tumor characteristics. Thus, the classifier is more discriminative than the baseline models like SSLW-CNN, DCNN, ResNet and MANet. Likewise, the recall values are always better than current methods indicating that the model can be used to detect more actual tumor cases. This enhancement is paramount in medical diagnosis, where false negative (overlooking a tumor) can be disastrous. The improved recall can be explained by the adversarial learning of DCGAN that permits the model to learn more complicated tumor patterns and rare variations of MRI images.

The trends of accuracy and F1-score also confirm the strength of the suggested framework. The gradually growing performance with the increasing dataset size designates that the model has a strong generalization ability. The HN-NAS component, unlike traditional deep learning models, dynamically reads network structures to find the best design to guarantee that the architecture is well adapted to the available dataset. The resulting automated optimization results in balanced increases in both precision and recall, and the subsequent increase in F1-scores. Dice Similarity Coefficient (DSC) findings establish the performance of this model in modeling spatial overlap of the predicted and actual tumor regions. The greater the DSC values, the better the quality of segmentation which is critical in accurate localization of tumor borders. This is because of the rich feature representations that are acquired in GAN-based training where the discriminator is able to draw meaningful structural and textual data in MRI scans. The next notable finding is a drastic decrease in the execution time. The DCGANHN-NAS model proposed is faster to infer than the traditional models. This is mainly because of the HyperNet mechanism which is effective in creating weights of many architectures without necessarily undergoing intensive training of each candidate model. The framework can be used in real-time clinical applications due to its high accuracy and computational efficiency. The role of every component is also confirmed with the help of the ablation study. With the independent application of DCGAN, the model enjoys more data diversity but inadequate architecture tuning. The application of NAS alone, on the other hand, enhances architecture choice but does not deal with data scarcity. The DCGAN-HN-NAS method is the most effective, which proves that both aspects are needed to achieve the highest quality of diagnostic results. Lastly, analysis results of the K-fold cross-validation indicate that there is very little deviation in accuracy among the folds, which means that the model is very stable and reliable. This proves that the suggested framework is not overfitted and generalizes to unknown data.

The performance analysis of the proposed DCGAN–HN-NAS technique establishes consistent and significant improvements across all evaluation metrics, confirming its efficiency for brain tumor classification and diagnosis. The precision results show a steady increase from 91.35% to 94.78% as the dataset size produces, representing a substantial reduction in false positive predictions due to enhanced feature learning from DCGAN-generated synthetic MRI images. Likewise, recall values of the model were good, development value was 96.98%, which is important for medical diagnosis in which the model was able to recognize tumor cases and to reduce the false negative cases as much as possible. The highest accumulated accuracy is 98.91%, indicating the strength of the integrated architecture in terms of the high classification accuracy of both tumor and non-tumor cases in all data distributions. The F1-score of 97.11% also demonstrates a balance between precision and recall, avoiding any tendency towards skewing the results. The Dice Similarity Coefficient is used to measure the spatial overlap between the predicted tumor regions and the ground truth annotations, with a value of 95.91% demonstrating a high degree of overlap and indicating that the model is effective in precisely localizing the tumor. Moreover, the execution time analysis points out that the suggested method significantly lowers the cost of computation related to existing methods due to the NAS mechanism of HyperNet, which generates optimal network weights without exploring all combinations. Integrating DCGAN with data augmentation and HN-NAS with automated architecture optimization yields a framework that is highly accurate, efficient and scalable, and demonstrates improvements over other state-of-the-art techniques in brain tumor analysis.

### Ablation study

To diagnose, locate and classify brain tumors, we conducted an ablation experiment using Deep Convolutional GAN (DCGAN) and Neural Architecture Search (NAS) based on HyperNet. Firstly, we removed the HyperNet module and tested the functioning of the DCGAN in generating synthetic data and identifying different types of brain tumor. Then we studied the impact of NAS on network optimization without the NAS service and compared it with the predefined architectures. The outcome indicates that HyperNet and NAS significantly improve the accuracy of classification, efficiency of the model, and the quality of the detection of the tumors, highlighting their relevance to providing the optimal diagnostic results.

### Influence of HN-NAS

The effectiveness of HN-NAS is due to its capability to automatically seek the best neural network structure. By utilizing a HyperNet to generate network weights for candidate architectures during the search process, HN-NAS significantly reduces the computational cost and time required compared to conventional architecture search methods. By automatically fine-tuning architectures to achieve more accuracy and performance than manually created models, this method improves the adaptability and optimization of deep learning models across a broad range of applications.

### Influence of the K-fold cross validation

The influence of K-fold cross-validation is significant in evaluating the stability, robustness, and generalization capability of the proposed DCGAN-HN-NAS model. By dividing the dataset into multiple folds and validating the model iteratively, the technique ensures that the model performs consistently across different data partitions. The obtained accuracy values ranging from 0.97 to 0.99, with a mean accuracy of 0.98, demonstrate that the proposed framework achieves reliable and stable classification performance with minimal variation among folds.

The results of the K-fold cross validation of the proposed DCGAN-HN-NAS model are shown in Table 8. The accuracy values achieved in different folds vary between 0.97 to 0.99, showing good stability and robustness of the proposed framework. The model has the highest accuracy of 0.99 in 3-fold, 8-fold and 10-fold validations and the lowest accuracy of 0.97 in 1-fold and 4-fold validations. The overall mean accuracy of 0.98 suggests that the proposed DCGAN-HN-NAS model achieves high and stable accuracy across various validation folds, showing that it can perform consistently and reliably.

**Table 8:** K-fold cross validation.

K-folds	DCGAN- HN-NAS Accuracy
**1-Fold**	0.97
**9-Fold**	0.98
**3-Fold**	0.99
**8-Fold**	0.99
**5-Fold**	0.98
**7-Fold**	0.98
**6-Fold**	0.98
**4-Fold**	0.97
**2-Fold**	0.98
**10-Fold**	0.99
**10-Fold Mean**	0.98

**Fig 13:**
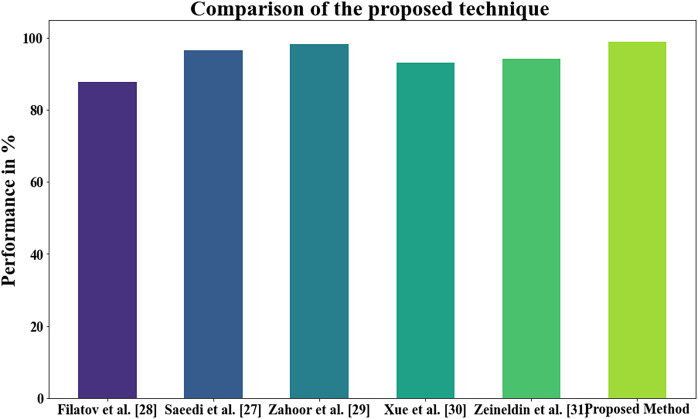
Comparison of the suggested technique with the other models.

Table 9 and Fig 13 provide a comparison of the suggested DCGAN-HN-NAS model with a number of contemporary state-of-the-art techniques across several datasets. The Res-BRNet model accomplishes a high accuracy of 98.22% when compared to models like EfficientNetB1 (87.67%), Multimodal CNNs (93.0% and 94.2%), and 2D CNN (96.45%). However, the proposed DCGAN-HN-NAS approach outperforms all the compared methods on T1-weighted contrast-enhanced MRI data, achieving the highest accuracy of 98.91%. This development can be attributed to the combined advantages of HyperNet-driven neural architecture optimization and DCGAN-based data augmentation, which improve both feature learning and the model’s generalization capability.

**Table 9:** Comparison of the suggested method with the other models.

Author	Technique	Dataset	Accuracy
**Filatov et al. [28]**	EfficientNetB1	Figshare , SARTAJ, Br35H4	87.67%
**Zahoor et al. [29]**	Res-BRNet (CNN)	Br35H, and figshare	98.22%
**Saeedi et al. [27]**	2D CNN	MRI dataset	96.45%
**Xue et al. [30]**	Multimodal CNN	Public dataset	93.0%
**Zeineldin et al. [31]**	Multimodal CNN	BraTS dataset	94.2%
**Proposed Method**	DCGAN- HN-NAS	T1-weighted contrast-enhanced MRI dataset	98.91%

### Challenges

The scarcity of large, highly annotated medical data, as well as the great disparity of MRI image quality due to the dissimilarity of scanners and scan settings, is the major challenge of brain tumor detection and classification. The heterogeneity of tumor size, shape and texture is further worsened by the fact that it is intrinsic and makes it difficult to classify and generalize the model. Also, most current deep learning models are not interpretable, which is not a good sign to be adopted in clinical practice. These issues underscore the necessity of efficient, scalable, and data-saving approaches to enhance diagnostic processes and provide consistency.

## Conclusion

The paper proposes a new approach to brain tumor diagnosis and classification based on a HyperNet-based Neural Architecture Search (NAS) network, along with Deep Convolutional Generative Adversarial Networks (DCGANs). The proposed approach outshines the other approaches in terms of efficiency and accuracy in detecting tumors. This method improves the training data and solves the problem of a lack of labeled medical images by generating high-quality images using DCGANs. The NAS enables HyperNet to explore different architectures of the neural network, which can be used to recognize the best ones depending on the specific neural network task of brain tumor diagnosis. This adaptive system not only improves the performance in classifications, but also provides an insight into the elements of architecture that facilitate the successful learning. The outcomes of this experiment show its feasibility in clinical settings in which rapid and accurate diagnosis is critical. Future studies ought to be aimed at testing the model in terms of its validation with the broader and more heterogeneous datasets, enhance the explanatory capabilities of the framework, and create more advanced attention mechanisms to drive more precise results. Also, it is important that the research in the future focuses on enhancing transparency and interpretability in the next-generation diagnostic technologies.

## References

[1] Sadoon TA, Ali MH. Deep learning model for glioma, meningioma and pituitary classification. Int J Adv Appl Sci. 2021;:88–98. doi: 10.11591/ijaas.v10.i1.pp88-98

[2] Devi OR, Bindu CS, Kumar ES. Identification And Classification Of Brain Tumor From MRI Using Transfer Learning Approach. In: 2022 International Conference on Computing, Communication, and Intelligent Systems (ICCCIS), 2022. p. 937–42. 10.1109/icccis56430.2022.10037223

[3] Nagaraj P, Muneeswaran V, Reddy LV, Upendra P, Vardhan Reddy MV. Programmed multi-classification of brain tumor images using deep neural network. In: 2020 4th International Conference on Intelligent Computing and Control Systems (ICICCS), 2020. p. 865–70. 10.1109/iciccs48265.2020.9121016

[4] Jans LBO, Chen M, Elewaut D, Van den Bosch F, Carron P, Jacques P, et al. MRI-based synthetic CT in the detection of structural lesions in patients with suspected sacroiliitis: comparison with MRI. Radiology. 2021;298(2):343–9. doi: 10.1148/radiol.2020201537 33350891

[5] Ammirati M, Nahed BV, Andrews D, Chen CC, Olson JJ. Congress of neurological surgeons systematic review and evidence-based guidelines on treatment options for adults with multiple metastatic brain tumors. Neurosurgery. 2019;84(3):E180–2. doi: 10.1093/neuros/nyy548 30629219

[6] Wahlang I, Maji AK, Saha G, Chakrabarti P, Jasinski M, Leonowicz Z, et al. Brain magnetic resonance imaging classification using deep learning architectures with gender and age. Sensors (Basel). 2022;22(5):1766. doi: 10.3390/s22051766 35270913 PMC8914787

[7] Alanazi MF, Ali MU, Hussain SJ, Zafar A, Mohatram M, Irfan M, et al. Brain tumor/mass classification framework using magnetic-resonance-imaging-based isolated and developed transfer deep-learning model. Sensors (Basel). 2022;22(1):372. doi: 10.3390/s22010372 35009911 PMC8749789

[8] Hu R, Hoch MJ. Application of diffusion weighted imaging and diffusion tensor imaging in the pretreatment and post-treatment of brain tumor. Radiol Clin North Am. 2021;59(3):335–47. doi: 10.1016/j.rcl.2021.01.003 33926681

[9] Chakraborty P, Das SS, Dey A, Chakraborty A, Bhattacharyya C, Kandimalla R, et al. Quantum dots: The cutting-edge nanotheranostics in brain cancer management. J Control Release. 2022;350:698–715. doi: 10.1016/j.jconrel.2022.08.047 36057397

[10] Biratu ES, Schwenker F, Ayano YM, Debelee TG. A Survey of Brain Tumor Segmentation and Classification Algorithms. J Imaging. 2021;7(9):179. doi: 10.3390/jimaging7090179 34564105 PMC8465364

[11] Kumar A, Manikandan R, Kose U, Gupta D, Satapathy SC. Doctor’s Dilemma: evaluating an explainable subtractive spatial lightweight convolutional neural network for brain tumor diagnosis. ACM Trans Multimedia Comput Commun Appl. 2021;17(3s):1–26. doi: 10.1145/3457187

[12] Allah AMG, Sarhan AM, Elshennawy NM. Edge U-Net: brain tumor segmentation using MRI based on deep U-Net model with boundary information. Expert Systems with Applications. 2023;213:118833. doi: 10.1016/j.eswa.2022.118833

[13] Filatov D, Ahmad Hassan Yar GN. Brain tumor diagnosis and classification via pre-trained convolutional neural networks. MedRxiv. 2022. doi: 10.1101/2022.07.18.22277779

[14] Sekhar A, Biswas S, Hazra R, Sunaniya AK, Mukherjee A, Yang L. Brain tumor classification using fine-tuned googlenet features and machine learning algorithms: IoMT enabled CAD system. IEEE J Biomed Health Inform. 2022;26(3):983–91. doi: 10.1109/JBHI.2021.3100758 34324425

[15] Rasool M, Ismail NA, Al-Dhaqm A, Yafooz WMS, Alsaeedi A. A novel approach for classifying brain tumours combining a squeezenet model with SVM and fine-tuning. Electronics. 2022;12(1):149. doi: 10.3390/electronics12010149

[16] Bibi N, Wahid F, Ali S, Ma Y, Abbasi IA, Alkhayyat A. A transfer learning based approach for brain tumor classification. IEEE Access. 2024. doi: 10.1109/ACCESS.2024.3425469

[17] Farzamnia A, Hazaveh SH, Siadat SS, Moung EG. MRI brain tumor detection methods using contourlet transform based on time adaptive self-organizing map. IEEE Access. 2023. doi: 10.1109/ACCESS.2023.3322450

[18] Abdelaziz Ismael SA, Mohammed A, Hefny H. An enhanced deep learning approach for brain cancer MRI images classification using residual networks. Artif Intell Med. 2020;102:101779. doi: 10.1016/j.artmed.2019.101779 31980109

[19] Rehman A, Naz S, Razzak MI, Akram F, Imran M. A deep learning-based framework for automatic brain tumors classification using transfer learning. Circuits Syst Signal Process. 2019;39(2):757–75. doi: 10.1007/s00034-019-01246-3

[20] Lee JH, Chae JW, Cho HC. Improved classification of different brain tumors in MRI scans using patterned-gridmask. IEEE Access. 2024. doi: 10.1109/ACCESS.2024.3377105

[21] Teng Q, Liu Z, Song Y, Han K, Lu Y. A survey on the interpretability of deep learning in medical diagnosis. Multimed Syst. 2022;28(6):2335–55. doi: 10.1007/s00530-022-00960-4 35789785 PMC9243744

[22] Brain tumor MRI dataset. [cited 2023 Oct 1]. https://www.kaggle.com/datasets/masoudnickparvar/brain-tumor-mri-dataset

[23] Bhimavarapu U, Chintalapudi N, Battineni G. Brain tumor detection and categorization with segmentation of improved unsupervised clustering approach and machine learning classifier. Bioengineering (Basel). 2024;11(3):266. doi: 10.3390/bioengineering11030266 38534540 PMC10967714

[24] Gao H, Zhang Y, Lv W, Yin J, Qasim T, Wang D. A deep convolutional generative adversarial networks-based method for defect detection in small sample industrial parts images. Applied Sciences. 2022;12(13):6569. doi: 10.3390/app12136569

[25] Han X, Li C, Wang Z, Liu G. NDARTS: a differentiable architecture search based on the Neumann Series. Algorithms. 2023;16(12):536. doi: 10.3390/a16120536

[26] Shaik NS, Cherukuri TK. Multi-level attention network: application to brain tumor classification. Signal, Image and Video Processing. 2022;16(3):817–24. doi: 10.1007/s11760-021-02022-0

[27] Saeedi S, Rezayi S, Keshavarz H, Niakan Kalhori R. MRI-based brain tumor detection using convolutional deep learning methods and chosen machine learning techniques. BMC Medical Informatics and Decision Making. 2023;23(1):16. doi: 10.1186/s12911-023-02114-6 36691030 PMC9872362

[28] Filatov D, Ahmad Hassan Yar GN. Brain tumor diagnosis and classification via pre-trained convolutional neural networks. MedRxiv. 2022. doi: 10.1101/2022.07.18.22277779

[29] Zahoor MM, Khan SH, Alahmadi TJ, Alsahfi T, Mazroa ASA, Sakr HA, et al. Brain tumor MRI classification using a novel deep residual and regional CNN. Biomedicines. 2024;12(7):1395. doi: 10.3390/biomedicines12071395 39061969 PMC11274019

[30] Xue J, Yao Y, Teng Y. Multi-modal tumor segmentation methods based on deep learning: a narrative review. Quantitative Imaging in Medicine and Surgery. Hong Kong, China: AME Publishing Company. 2022. 10.21037/qims-23-818 PMC10784092 38223046

[31] Zeineldin RA, Karar ME, Burgert O, Mathis-Ullrich F. Multimodal CNN networks for brain tumor segmentation in MRI: A BraTS 2022 challenge solution. In: Proceedings of the International MICCAI Brainlesion Workshop, Singapore; 2022. p. 127–37.

